# Endometrial carcinoma in a young subfertile woman with polycystic ovarian syndrome

**DOI:** 10.4103/0974-1208.63122

**Published:** 2010

**Authors:** K Jayakrishnan, R Anupama, Aby Koshy, R Raju

**Affiliations:** Department of Reproductive Medicine, Fertility Research and Gynaec Centre, KJK Hospital, Trivandrum, Kerala 695 015, India

**Keywords:** Endometrial adenocarcinoma, infertility, polycystic ovaries

## Abstract

Adenocarcinoma of the endometrium is a morbid condition in women under 40 years of age with an incidence of 25%. However, patients with anovulatory polycystic ovarian syndrome are at risk of developing endometrial carcinoma. The disease is often advanced when diagnosed, thereby depriving the woman of the option for fertility sparing conservative approach. In young women with menstrual abnormalities and polycystic ovarian disease and/or infertility, an endometrial evaluation should be performed. Carcinoma endometrium should be kept in mind while evaluating young women with polycystic ovary syndrome for abnormal uterine bleeding. Only strictly selected patients should, therefore, be indicated for long-term progestogen treatment and careful evaluation before and after treatment should be performed.

## INTRODUCTION

An association between polycystic ovary syndrome (PCOS) and endometrial carcinoma was first suggested in 1949. However, obesity, hyperinsulinemia, and hyperandrogenism, which are also the features of PCOS, are risk factors for endometrial carcinoma, but it does not necessarily follow that the incidence or mortality from endometrial cancer is increased in women with the syndrome.[1–3] Lack of clinical suspicion and reluctance to do an endometrial evaluation may delay this rare diagnosis of endometrial cancer in young women. This is highlighted in this case report where an advanced endometrial cancer was encountered in a young woman with infertility.

## CASE REPORT

A 31-year-old woman with a 4-year history of primary subfertility was referred to the assisted conception unit for investigation and treatment. Her menarche had been at the age of 14 years and her menstrual cycles were irregular.

**Figure 1:**
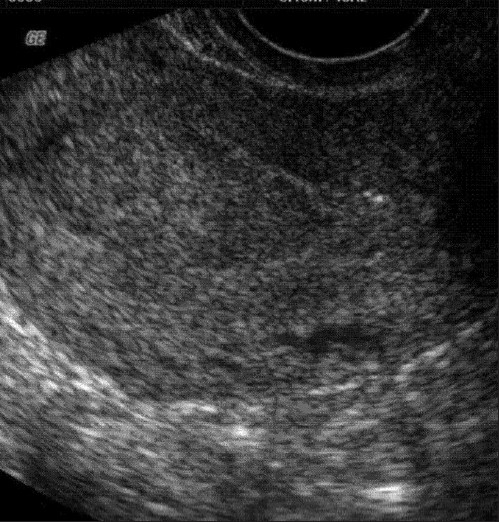
Ultrasound showing thickened endometrium

**Figure 2:**
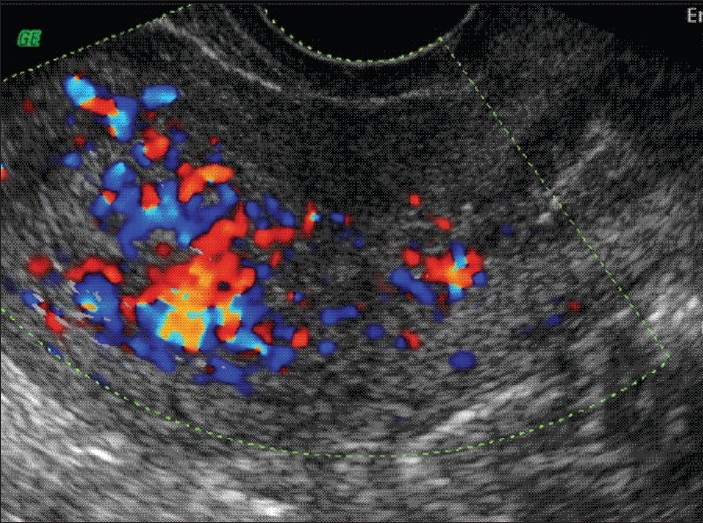
Doppler showing increased endometrial vascularity

On examination, she was neither hirsute nor obese with a body mass index of 23 (in Asian women, normal range is 18.5-23 kg/m^2^).[4] Abdomino-pelvic examination was normal and her recent cervical smear test had been negative. Blood tests for thyroid function (T_3_ 140 ng/dl, T_4_ 8.3 μg/dl, TSH 3.5 mIU/ ml) and prolactin (12 IU/ml) concentrations were normal. Her GTT values were within normal limits (98/135/124/112 mg/dl). She was normotensive (blood pressure of 122/80 mmHG). Her lipid profile was found to be normal (total cholesterol 182 mg/dl, triglycerides 120 mg/dl, HDL 63 mg/ dl, LDL 92 mg/dl, VLDL 24 mg/dl). A transvaginal ultrasound scan showed an endometrial thickness of 12.3 mm in secretory phase of cycle with PCO-type ovaries. Her husband was found to be mild oligoasthenospermic. Clomiphene citrate/gonadotrophin-induced ovulations were also unsuccessful in achieving a conception after three attempts. Preoperative transvaginal sonogram showed polycystic ovaries with an endometrial thickness of 18.7 mm. Operative laparoscopy combined with hysteroscopic evaluation was done, which revealed polycystic ovaries with minimal endometriosis, and a hyperplastic endometrium. Laparoscopic ovarian drilling was done and an endometrial biopsy was performed. Histopathology report was consistent with hyperplastic endometrium with squamous/morular metaplasia. She was put on continuous progestogen for a period of 3 months and advised to be on strict follow-up. Unfortunately, she did not turn up. After 1 year, she presented with complaints of irregular bleeding per vaginum. Transvaginal sonography showed an endometrial thickness of 23 mm with increased vascularity on Doppler evaluation [Figures 1 and 2].

**Figure 3:**
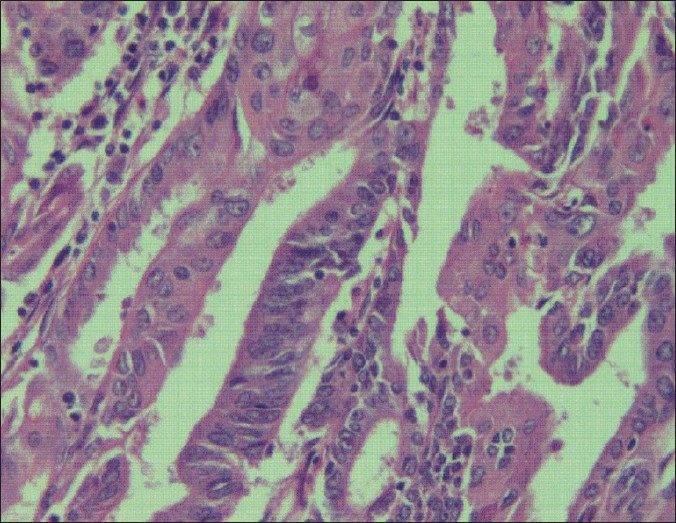
Histopathology of endometrioid adenocarcinoma

Repeat endometrial biopsy was consistent with well-differentiated adenocarcinoma endometrium [Figure 3]. Considering the rarity expert opinion gathered from an oncology center gave the impression as low grade aggressive endometrial adenocarcinoma with early invasive features with no normal endometrium favoring implantation. The treatment options like long-term, high- dose progesterones and follow-up endometrial biopsy were explained to her. Similarly, the chance of early stage endometrial carcinoma progressing to invasive type was explained to the patient. The role of surgery and its benefits and demerits were thoroughly explained to the couple. Ample time was given to the couple to come to a decision. They decided to proceed with laparotomy. At laparotomy, the uterus and ovaries appeared normal. There was no free fluid in the pelvis. The pelvic and para-aortic lymph nodes were not enlarged. A total abdominal hysterectomy with bilateral salpingo-oophorectomy with pelvic lymph node sampling was performed. Histopathology was consistent with well-differentiated adenocarcinoma endometrium stage 1 grade 1. Hence she did not require any further treatment.

## DISCUSSION

Endometrial carcinoma in young nulliparous women poses a challenge for the diagnosis and management. This case report illustrates that young subfertile women with PCOS are at risk of developing endometrial carcinoma. Moreover, this case report highlights the dilemma of various treatment options for early disease in such patients. Physicians in general and fertility experts in particular must be vigilant for signs of endometrial disease in these young women. Signs and symptoms such as treatment failure, abnormal vaginal bleeding, or unprovoked bleeding encountered at the time of attempted embryo transfer should be thoroughly investigated since cancer could be present during infertility treatment.

One of the well-documented effects of estrogen on the endometrium is its growth-stimulating effect, which can produce a progression of changes from benign proliferation to atypical hyperplasia and adenocarcinoma.[5–7] Obesity has strong association with PCOS. Obesity increases the risk to two- to threefold if an individual is 50 lb more than the ideal weight for that person. Moreover, two- to threefold increased risk has been documented in nulliparous women. Anovulation due to unopposed estrogens contributes to this situation. The endometrium of PCOS is unusually prone for malignancies. Literature suggests several other theories. Since androgen receptors and steroid receptor coactivators are overexpressed in PCOS, biomarkers like alpha(v) beta 3 integrin and glycodelin are decreased in PCOS. Epithelial expression of estrogen receptor alpha is abnormal in the window of implantation in PCOS.[8] On the other hand, progesterone induces regular sloughing of the endometrium, thereby removing endometrial tissue that might otherwise become hyperplastic. Furthermore, progesterone can reverse various degrees of hyperplasia to normal endometrial histology also. Endometrial morphological changes with progestogens vary from the suppression of endometrial glandular growth, through stromal decidualization and leukocytic infiltration to glandular atrophy and stromal focal necrosis. Due to prolonged treatment, connective tissue fibers increase to some degree and may be accompanied by endometrial fibrosis and calcification. Clinical and histological data have demonstrated that all these changes, including fibrosis and calcification, return to normal in a short period after discontinuing the treatment.[9]

The standard treatment for endometrial carcinoma is total abdominal hysterectomy with bilateral salpingo-oophorectomy. In young women with low histological grade and early stage of the disease, conservative hormonal therapy has been tried with close follow-up. There are reports of high-dose medroxyprogesterone acetate (600 mg/ day) treatment with endometrial evaluation in every 3 months to assess the effects of medication. If the response is not satisfactory, hysterectomy is advocated. For a successful outcome following conservative approach, a strict clinical staging in the form of physical examination and imaging with ultrasound, CT or MRI, and a cautious evaluation of histological grading by a pathologist are required. This is then followed by at least 6 months of progestogen therapy and evaluation of response by endometrial sampling.[10–12] There are reports of conservative hormonal therapy with megestrol acetate and antiestrogens like Tamoxifen for 6 months followed by hysteroscopic guided endometrial sampling and the help of laparoscopic tubal clamping. This will be followed by a combination of contraceptive pills to ensure withdrawal bleeding.[13] However, Daniel *et al.*,[14] have reported high incidence of endometrial hyperplasias with Tamoxifen. There are reports of endometrial carcinoma progressing to advanced stages in those treated conservatively with Megace and levonorgestrel intrauterine device. Women with endometrial cancer who want fertility preservation should be counseled regarding the possible risk of advanced disease if surgical therapy is delayed. There are no randomized controlled data to guide conservative therapy.[15]

Reproductive options remain a meaningful quality-of-life goal even for the patients with cancer. Literature favors routine pelvic MRI for the follow-up of endometrial cancer patients who retain their uterus. Hysteroscopy and dilation, and curettage alone may not be sufficient since chance of occult myometrial recurrence is more.[16] Conservative therapy is feasible only in carefully selected young women with endometrial cancer. Recurrence rates were high during long-term observations even after pathologically complete remissions. Therefore, close follow-up is recommended.[17] We did not consider such conservative management with progestogen in our patient second time for the following reasons:

- The conversion of hyperplasia to frank malignancy was fast in her case.
- She had poor compliance.

This case highlights the need for endometrial sampling in young women with anovulatory cycles so that hyperplasia can be diagnosed and treated before frank invasion. Even if invasive disease is diagnosed early, at least fertility-sparing conservative hormonal therapy can be tried in highly motivated and selected patients only.
